# The Novel PPAR α/γ Dual Agonist MHY 966 Modulates UVB–Induced Skin Inflammation by Inhibiting NF-κB Activity

**DOI:** 10.1371/journal.pone.0076820

**Published:** 2013-10-09

**Authors:** Min Hi Park, Ji Young Park, Hye Jin Lee, Dae Hyun Kim, Ki Wung Chung, Daeui Park, Hyoung Oh Jeong, Hye Rim Kim, Chan Hum Park, So Ra Kim, Pusoon Chun, Youngjoo Byun, Hyung Ryong Moon, Hae Young Chung

**Affiliations:** 1 Molecular Inflammation Research Center for Aging Intervention (MRCA), College of Pharmacy, Pusan National University, Busan, Korea; 2 Laboratory of Biochemistry, Pusan National University, Busan, Korea; 3 Laboratory of Medicinal Chemistry, College of Pharmacy, Pusan National University, Busan, Korea; 4 College of Pharmacy, Inje University, Gimhae, Gyeongnam, Korea; 5 College of Pharmacy, Korea University, Chungnam, Korea; Virginia Tech, United States of America

## Abstract

Ultraviolet B (UVB; 290~320nm) irradiation-induced lipid peroxidation induces inflammatory responses that lead to skin wrinkle formation and epidermal thickening. Peroxisome proliferator-activated receptor (PPAR) α/γ dual agonists have the potential to be used as anti-wrinkle agents because they inhibit inflammatory response and lipid peroxidation. In this study, we evaluated the function of 2-bromo-4-(5-chloro-benzo[d]thiazol-2-yl) phenol (MHY 966), a novel synthetic PPAR α/γ dual agonist, and investigated its anti-inflammatory and anti-lipid peroxidation effects. The action of MHY 966 as a PPAR α/γ dual agonist was also determined in vitro by reporter gene assay. Additionally, 8-week-old melanin-possessing hairless mice 2 (HRM2) were exposed to 150 mJ/cm^2^ UVB every other day for 17 days and MHY 966 was simultaneously pre-treated every day for 17 days to investigate the molecular mechanisms involved. MHY 966 was found to stimulate the transcriptional activities of both PPAR α and γ. In HRM2 mice, we found that the skins of mice exposed to UVB showed significantly increased pro-inflammatory mediator levels (NF-κB, iNOS, and COX-2) and increased lipid peroxidation, whereas MHY 966 co-treatment down-regulated these effects of UVB by activating PPAR α and γ. Thus, the present study shows that MHY 966 exhibits beneficial effects on inflammatory responses and lipid peroxidation by simultaneously activating PPAR α and γ. The major finding of this study is that MHY 966 demonstrates potential as an agent against wrinkle formation associated with chronic UVB exposure.

## Introduction

Peroxisome proliferator-activated receptors (PPARs) belong to the nuclear receptor superfamily, a family of ligand-activated transcriptional factors. PPARs function as ligand-dependent transcription factors and can heterodimerize with retinoid X receptors and then bind to PPAR-responsive elements (PPRE) in target gene promoters, which usually leads to transcriptional activation. Another function of PPARs is the inhibition of inflammatory gene expression. In several model systems, PPARs repressed the target genes of nuclear factor-κB (NF-κB). Another function of PPARs is the inhibition of inflammatory reaction [1]. For example, tesaglitazar, a well known PPAR α/γ dual agonist, has been reported to reduce pro-inflammatory cytokine levels [2] although its effects on wrinkle formation are unknown.

The major function of the epidermis is to provide a defense against physical environmental pollutants and UVB [3]. These environmental toxicants are inherent oxidants and/or directly or indirectly drive the production of a variety of reactive oxidants also known as reactive oxygen species (ROS), such as, superoxide, hydrogen peroxide, and the hydroxyl radical [4]. ROS have an established role in UV-induced skin aging, which is characterized by wrinkle formation. In general, wrinkles are created by alterations in the dermal matrix, whereby collagen levels are reduced by accelerated breakdown and collagen synthesis is reduced [5].

UVB irradiation can have direct and indirect adverse biologic effects, which include the induction of oxidative stress, DNA damage, and premature skin aging [6]. Furthermore, UVB-induced ROS enhance inflammatory response by activating NF-κB [7]. In addition, UVB enhances the levels of NF-κB responsive proteins, such as, inducible nitric oxide synthase (iNOS) and cyclooxygenase-2 (COX-2), and induces the production of nitric oxide (NO), which plays a central role in regulation of skin cell apoptosis [8–10]. NO is produced from L-arginine and oxygen in a reaction catalyzed by iNOS and causes lipid peroxidation when it is transformed into cytotoxic peroxynitrite (ONOO^-^) by reacting with ROS [11,12]. Thus, the damage of skin tissues by lipid peroxidants is responsible for the wrinkle formation that is indicative of photoaging [4].

In the previous study, we reported that MHY 966 suppresses melanogenesis by inhibiting the generation of NO [13]. This study was undertaken to identify a novel PPAR α/γ dual agonist and to explore the hypothesis that MHY 966 prevents UVB-induced collagen degrdation by inhibiting inflammatory response. In the present study, we identified a novel PPAR α/γ dual agonist MHY 966, by using a reporter gene assay and by docking simulation. Furthermore, the anti-inflammatory effects of MHY 966 were explored in UVB-induced HRM2 mice. Based on these results, it appears that MHY 966 activates both PPAR α and γ, and alleviates inflammatory response, making it a potentially new treatment for UVB-induced skin inflammation.

## Results

### MHY 966 increased the transcriptional activities of PPAR α and γ

**Figure 1:**
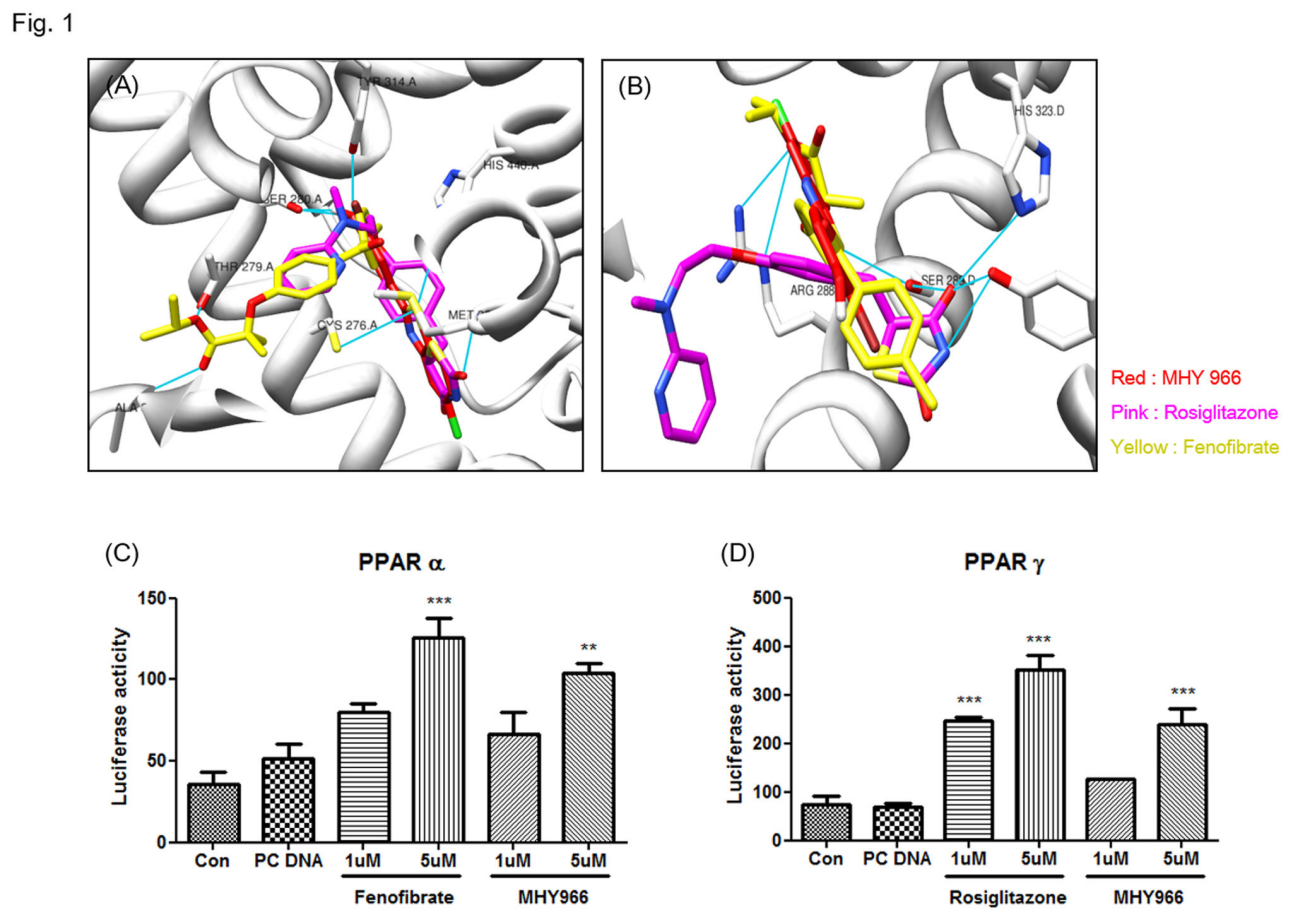
MHY 966 functioned as a PPAR α/γ dual agonist. (A) and (B), Docking simulation was performed to identify interaction between LBD and MHY 966. Docking modes of MHY 966 on the LBD of human PPAR α and γ. MHY 966 has similar binding sites compared with known PPAR α and γ agonist, fenofibrate and rosiglitazone, respectively. For luciferase assay, AC2F cells overexpressing PPAR α (C) and PPAR γ (D) were treated with 1 or 5 μM fenofibrate, rosiglitazone, or MHY 966 for 6 h. Bars represents means ± SEMs of triplicates results. *** P < 0.001, ** *P* < 0.01 and * *P* < 0.05 versus cell treated with PC DNA group. C and D, .

For specific interactions between nuclear hormone receptors and their ligands, one of the key chemical bonds is the hydrogen bond, which often links ligands and amino acid residues in the ligand domain of nuclear hormone receptors. To identify a novel PPAR α/γ dual agonist, we used the Autodock 4.2 program. According to Autodock 4.2, MHY 966 linked with a 2-bromo phenol to provide numerous hydrophobic interactions in the binding pocket as well as same binding pocket with fenofibrate and rosiglitazone, known as PPAR α and γ positive control, respectively (Figure 1 A and B). The binding energies of MHY 966 were -9.91 kcal/mol whereas fenofibrate were -8.80 kcal/mol in PPAR α, in another case PPAR γ was -7.80 kcal/mol whereas rosiglitazone was -8.03 kcal/mol. To confirm the specificity and sensitivity of MHY 966 in the regulation of transcriptional activities of PPARs, reporter gene assay were performed. As shown in Figure 1C and D, MHY 966 increased PPAR α and γ in a dose dependent manner in AC2F rat liver cells. 

### MHY 966 inhibited UVB-induced collagen digestion in HRM2 mice

**Figure 2:**
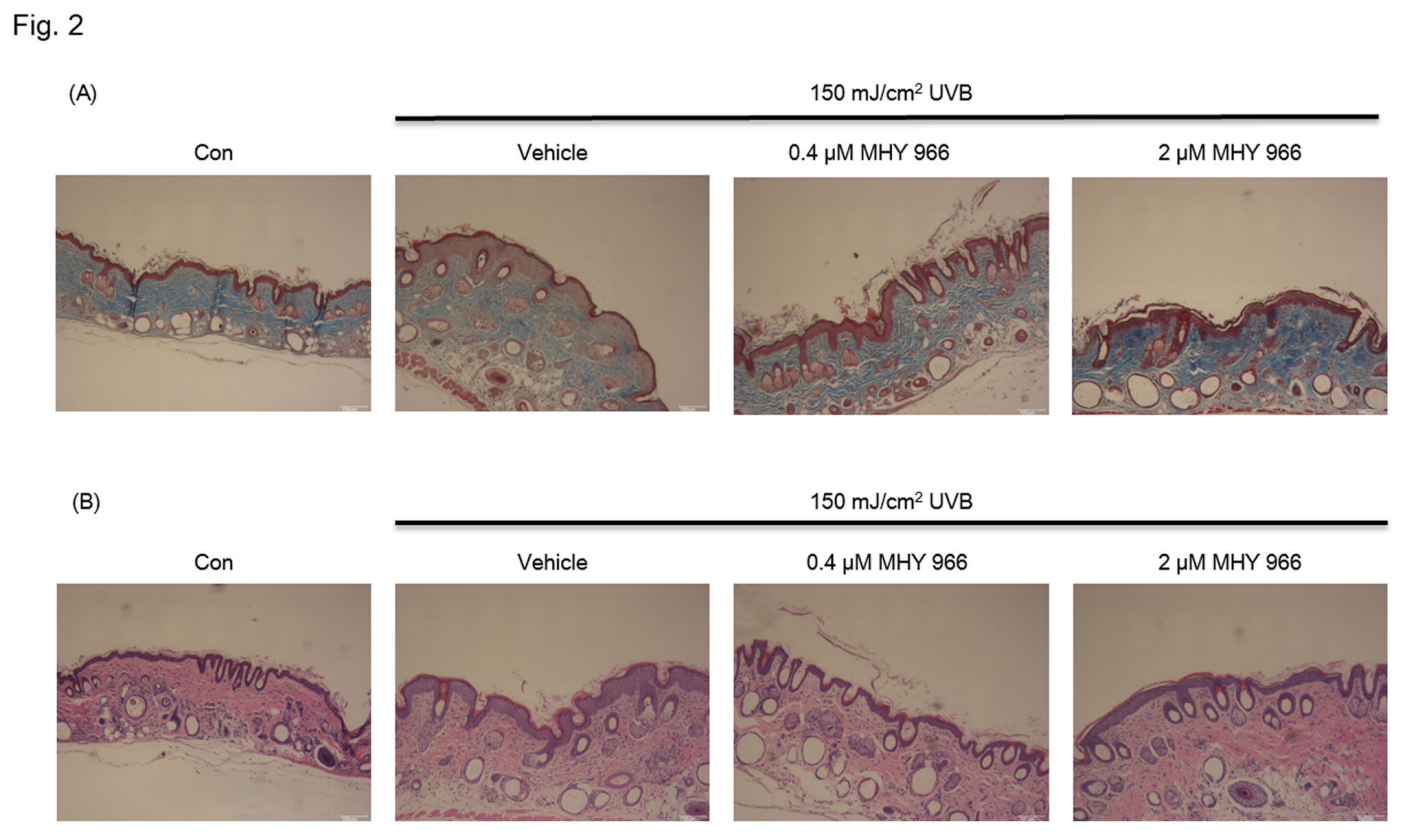
Effects of the topical administration of MHY 966 on UVB-induced epidermal thickness and collagen destruction in HRM2 mouse dorsal skin. Histological sections of mouse dorsal skins were stained with Masson-trichrome for dermal collagen fibrates (A). Paraffin sections were photographed after H&E staining for UVB induced skin damage (B). Original magnification: 200 X.

Histological staining with Masson-trichrome for dermal collagen fibrates showed that exposure UVB reduced cellular collagen levels in the intra dermis, indicating elevated digestion of dermal connective tissues. In contrast, strong staining of collagen fibers was observed in MHY 966 treated HRM2 mice exposed to UVB as compared with that observed in UVB alone exposed mice (Figure 2A).

Skin epidermal thickening was measured in UVB irradiated HRM2 mice by H&E staining, and epidermal thickness of dorsal skin was found to be significantly increased by UVB. On the other hand, the topical application of MHY 966 inhibited this UVB induced increase in epidermal thickness. Accordingly, topical treatment with MHY 966 was found to have protective effects on UVB induced skin damage (Figure 2B).

### MHY 966 reduced pro-inflammatory protein expression in UVB induced HRM2 mice

**Figure 3:**
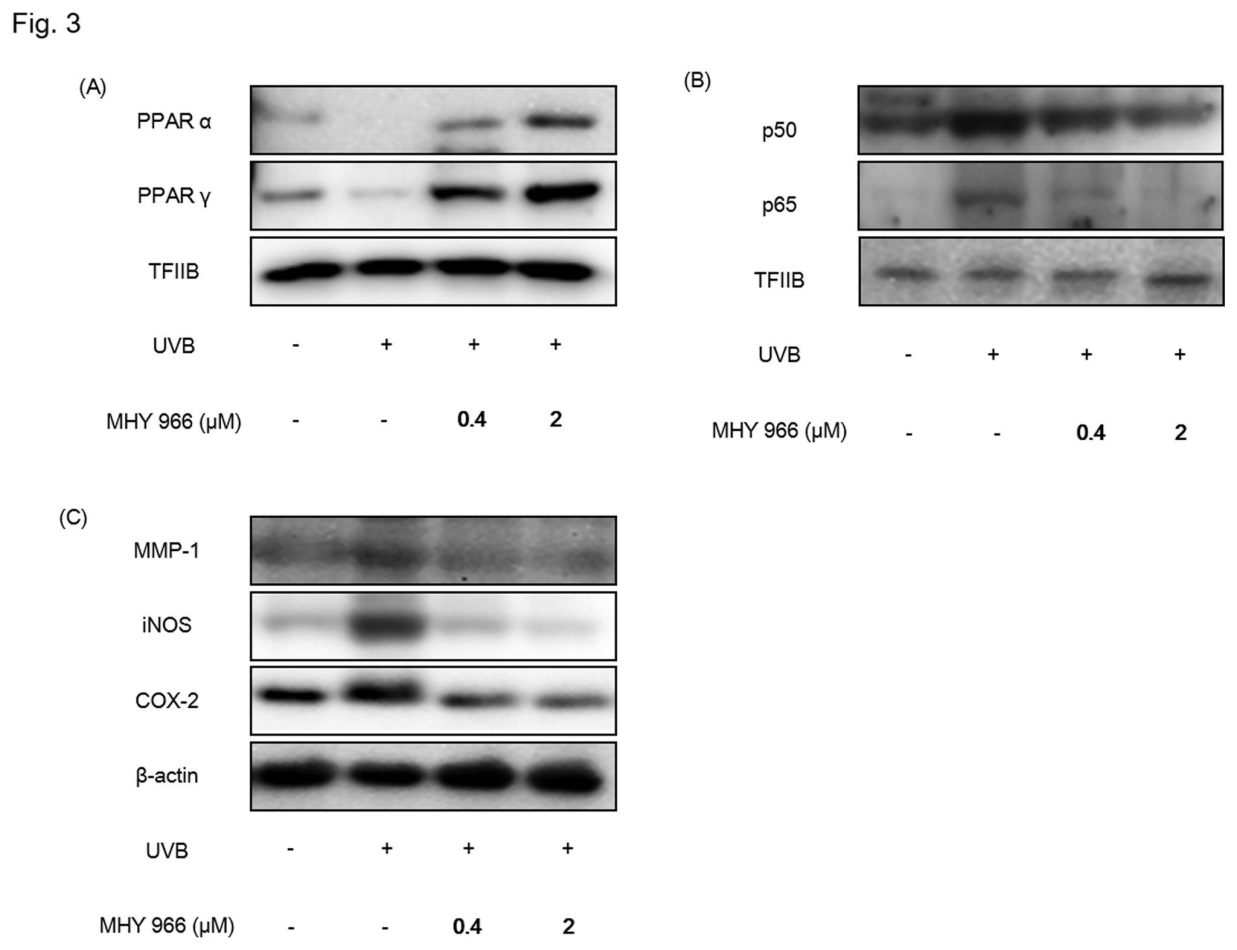
MHY 966 both activated PPAR α and γ, and inhibited inflammatory responses in UVB-induced HRM2 mouse skins. Western blot analysis was performed to detect the levels of PPAR α PPAR γ (A), NF-κB (B), MMP-1, COX-2 and iNOS (C) in skin hommogenate. TFⅡB and β-actin blots were shown to clarify the same amount of protein loaded in nuclear and cytosolic fraction, respectively. 0.4 or 2 μM of MHY 966 was topically applied on dorsal skin daily at for 17 days and then exposed to UVB using a BEX-800 UVB lamp (UltraLun, Claremont, Ca, USA) at 150 mJ/cm^2^.

Previous studies have indicated that pro-inflammatory response is a causative factor of wrinkle formation and skin aging [14]. In the present study, we examined the effect of MHY 966 on the inflammatory responses to UVB in dorsal skin by activating PPAR α and PPAR γ. Western blot analysis shows that PPAR α and PPAR γ expression levels increased in MHY 966 treated mice skin (Figure 3 A). Indeed, while UVB treated mice showed elevated levels of NF-κB, and NF-κB mediated inflammatory factors, such as metalloprotease-1 (MMP-1), iNOS and COX-2 as compared with normal control mice, treatment with MHY 966 reduced these expressional elevations (Figure 3 B, C). 

### MHY 966 improved lipid peroxidation by inhibiting ONOO^-^ generation

**Figure 4:**
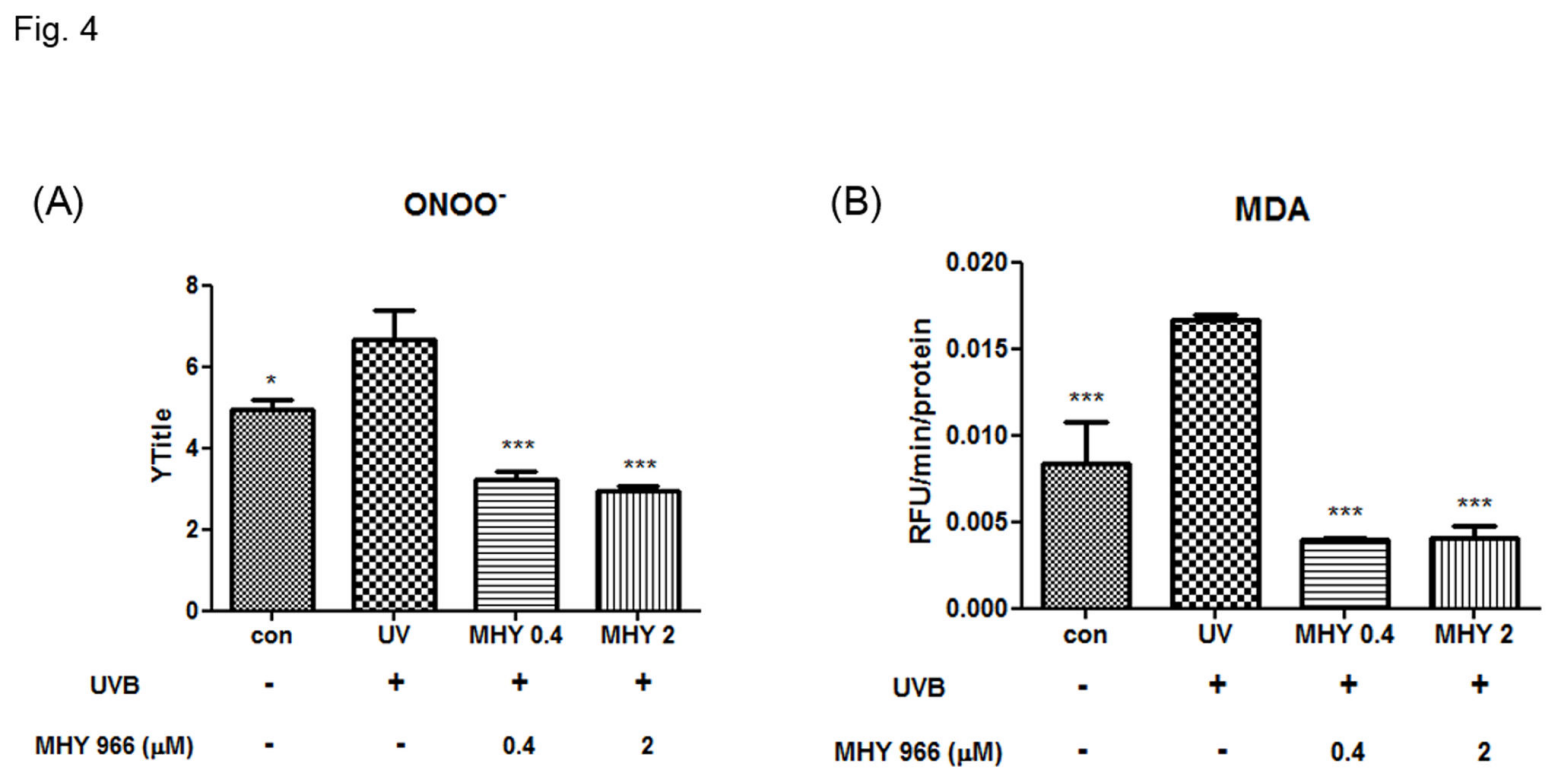
Effects of MHY 966 on UVB-induced ONOO^-^ and MDA in HRM2 mouse dorsal skin. ONOO^-^ was measured by DHR-123 with a fluorescent probe (A) and MDA was measured by TBARS assay (B) in HRM2 mouse dorsal skin. Bar represent means ± SEMs of six mice and normalized mg/protein. *** P < 0.001, ** *P* < 0.01 and * *P* < 0.05 versus UVB-exposed mice.

NO is synthesized by iNOS and generates highly reactive ONOO^-^. Thus, we examined whether MHY 966 suppresses ONOO^-^ generation. In accordance with previous reports, ONOO^-^ interacted with lipid-rich plasma membranes and caused lipid peroxidation. Previous research indicated that UVB produces ONOO^-^ and that this causes lipid peroxidation. In the present study, whereas UVB induced mice showed higher levels of ONOO^-^ than normal control mice, treatment with MHY 966 reduced ONOO^-^ levels (Figure 4A). In addition, MHY 966 effectively reduced UVB-induced increases in MDA levels. (Figure 4B)

## Discussion

A number of authors have reported that UVB causes serious oxidative damage in skin, because the polyunsaturated fatty acids of cellular membrane are attacked by lipid peroxides [4,5]. Skin photoaging is characterized by the degradation of collagen which leads to enhanced wrinkle formation [15] via expression of Matrix metalloproteases (MMPs). MMPs are important players in UVB induced skin diseases including psoriasis and cancer, and aging. 

The present studies have showed that PPARs regulate important cellular functions, including cell proliferation, differentiation, and inflammation. The anti-inflammatory effects of PPARs are important because pro-inflammatory responses play key roles in UVB induced skin aging. In particular, PPAR signals have been demonstrated to play a role in skin development and barrier formation [16,17]. In the present study, we found that MHY 966 activates PPAR α and γ, but the potency was less than that of fenofibrate, a PPAR α agonist, and rosiglitazone, a PPAR γ agonist, in in vitro transcriptional activity (Figure 1). However, this result conflict with the conclusion in our previous study [13]. In our previous study, we performed the Lantha Screen TR-FRET PPAR γ competitive binding assay. It just shows the interaction of an agonist with the ligand binding domain of PPAR γ. In this study, however, we conducted luciferase assay in AC2F cells. In cell culture systems, the results may vary depending on a variety of conditions. Thus, the conflict of conclusion happened between the previous and present studies, because of the different types of the experiments.

In addition, MHY 966 was found to be a more potent agonist of PPAR α than PPAR γ. Furthermore, PPAR γ agonist like rosiglitazone and pioglitazone, typically possess a small polar region and a hydrophobic region that form hydrogen bonds and hydrophobic interactions, respectively, with LBD (ligand binding domain) [18]. Hydrogen bonding typically occurs between His323, Tyr473, and His289 of PPAR γ LBD and carbonyl oxygens of the ligand. Hydrogen bonding of the ligand to Tyr473 is critical for stabilization of the AF-2 region of name [19]. However, MHY 966 does not form a hydrogen bond with Tyr473, which is probably why binding between PPAR γ and MHY 966 is weak compared with rosiglitazone. 

MHY 966 activates PPAR α and γ, but the potency was less than that of fenofibrate, a PPAR α agonist, and rosiglitazone, a PPAR γ agonist, in in vitro transcriptional activity. However, it is necessary to use MHY 966 instead of fenofibrate or rosiglitazone for their side effects [20]. In addition, PPAR γ related side effects can be minimized by the combination of PPAR γ potency and PPAR α potency equal to or more than the PPAR γ potency [21]. Furthermore, because the synthetic PPAR α and γ dual agonist independently ameliorates inflammation, the PPAR α/γ dual agonist might be even more effective.

UVB radiation is known to damage DNA directly and indirectly and to perturb extracellular matrix homeostasis by increasing MMP activity [22]. Histologic and ultrastructural studies have shown that photodamaged skin is associated with increased epidermal thickness and alterations in connective tissue organization [23]. Furthermore, in one study, MMP activity was increased in skin exposed to UVB, and this upregulation led to collagen destruction and photoaging [24]. In addition, in this previous study, it was shown that UVB augmented collagen degradation in dermal fibroblasts probably via activated collagenolytic MMP. On the other hand, the present study shows that MHY 966 inhibits UVB-induced MMP-1 overexpression and collagen degradation by suppressing NF-κB activation (Figure 3). This inhibition is probably related to the effect of PPARs on NF-κB-mediated transactivation exhibits because NF-κB activity is essential for MMP-1 expression [25], and PPARs could interfere with the NF-κB pathway via reducing NF-κB binding activity [26] and physically interacting with both p65 and p50 [27]. Therefore, these findings support the view that MHY 966 could inhibit inflammatory response via direct or indirect interfere with NF-κB.

UVB exposure leads to DNA damage and the formation of ROS, induces markers of inflammatory response like COX-2 and iNOS by activating NF-κB, and damages the integrity of extracellular matrix [28]. UVB has also been shown to upregulate iNOS expression to produce NO at high enough levels to react with superoxide to form ONOO^-^ and initiate lipid peroxidation processes [29]. In fact, lipid peroxidation induced by oxidative stress is a cause of the mass of keratinocytes, the cellular membranes of which contain considerable amounts of unsaturated lipid and cholesterol. In a previous study, we found that MHY 966 treatment reduces NO level increases caused by UVB exposure [13]. On the other hand, we found MHY 966 suppressed UVB-induced inflammatory response, including COX-2 and iNOS, by inhibited not ROS generation (data not shown) but NF-κB activation (Figure 3), thus we supposed that MHY 966 attenuated the UVB-induced overexpression of ONOO^-^ , and inhibited lipid peroxidation processes (Figure 4). These findings indicate MHY 966 inhibits epidermal thickening of dorsal skin by inhibiting UVB-induced inflammatory responses.

Summarizing, we found that the novel PPAR α/γ dual agonist, MHY 966 favorably affects collagen degradation caused by skin photoaging by activating PPAR α and γ, and thus, inhibits inflammatory responses. Therefore, we suggest PPAR α/γ dual agonists, be considered potential agents that inhibit wrinkle formation and skin photoaging, and that MHY 966 be viewed as a potential lead compound for the development of anti-skin aging agents.

## Materials and Methods

### Docking simulation of PPARs and target compounds

The crystal structure of PPAR α/γ were extracted from the PDB archives (entry code PPAR α: 1K7L, PPAR γ: 3DZY) [30] and employed as the target in docking calculations. For docking simulation, we used AutoDock4.2 program [31] which is the most commonly used because of its automated docking capability [12] among the many tools available for *in silico* protein-ligand docking. Because AutoDock program uses a grid-based method to allow rapid evaluation of the binding energy of trial conformations, we computed a grid box of the docking pocket on PPAR α/γ using the AutoGrid4 included in the Autodock4.2 program. The binding pocket defined as the grid box made of 40×40×40 points with grid spacing of 0.375 Å. The binding pocket was centered on the predefined active sites in human PPAR α and γ structures which got from PDB database. We performed docking simulations between PPAR α/γ and MHY 966, or with a fenofibrate (a rosiglitazone), which was used as the reference inhibitor. To prepare compounds for docking simulation, we performed the following steps: (1) 2D structures were converted into 3D structures, (2) charges were calculated, and (3) hydrogen atoms were added using the ChemOffice program (http://www.cambridgesoft.com).

### Shared pharmacophore of target compounds

A pharmacophore is an ensemble of ligand features required for interaction with a specific receptor in biological response [32]. The pharmacophore model was generated using the LigandScout 3.0 program [33]. Based on atom types, the chemical features of target compounds were defined in terms of pharmacophore elements, such as, hydrogen bond acceptors, hydrogen bond donors, positive ionizable areas, negative ionizable areas, hydrophobic interactions, and aromatic rings. 

### Transfection and luciferase assay

For luciferase assays, 0.1 ug of plasmid was transfected into 5 x 104 cells per well seeded in a 24-well plate in 500 μl of DMEM supplemented with 5% FBS at 37 °C in a humidified 95% air/5% CO2 atmosphere. Cells were transfected with lipofectamine transfection reagent and the plasmids used for transfection with 3xAOX-TK-luciferase reporter vector. After 12 h of transfection, cells were washed, and treated with MHY 966, fenofibrate, or rosiglitazone for 6 h and luciferase activities were then detected using the One-Glo Luciferase Assay System (Promega, USA). Luciferase activities were measured using a TECAN GENios luminescence plate reader (Tecan Instruments, Salzburg, Austria)

### Animals and skin characteristics

Male, 4-week old, HRM2 mice were obtained from the Hoshino Laboratory (Saitama, Japan) and maintained under a 12 h light/dark cycle at 23 ± 1°C and 50 ± 5% RH under specific pathogen-free conditions on a standard diet (Superfeed Co. South Korea) ad libitum. After acclimatization for 1 week, mice were randomly divided into 5 groups of 6 animals. MHY 966 was dissolved in vehicle consisting of ethanol and propylene glycol (3:7 vol/vol), and then topically applied to a designated 2 cm^2^ X 2 cm^2^ site on dorsal skin daily for 17 days. Mice were exposed to UVB using a BEX-800 UVB lamp (UltraLun, Claremont, Ca, USA) at 150 mJ/cm^2^. A photograph of the dorsal skin of each mouse was taken using a digital camera (Canon, Japan), just prior to sacrifice. The animal protocol used in this study has been reviewed by the Pusan National University-Institutional Animal Care and Use Committee (PNU-IACUC) on their ethical procedures and scientific care, and it has been approved (Approval Number PNU-2012-0102).

### Histology and microscopy

For histological analyses, skins were fixed in 10% formalin at room temperature. Paraffin-embedded skin specimens were sectioned at 5 μm, deparaffinized, and stained with Masson-trichrome to visualize collagen fibers and stained with hematoxylin and eosin (H&E) for light microscopic evaluation. Staining tissue sections were examined under an optical microscope (Eclipse TS100; Nikon Instruments Inc., Melville, NY, USA), and five or six photographs (200 X) were taken per section. Epidermal thickness was defined as the distance from the basal layer to the stratum granulosum/stratum corneum junction. Thicknesses were measured in each photograph at 10 random sites. 

### Quantification of ONOO^-^ and malondialdehyde (MDA) in skin homogenates

ONOO^-^ generation was measured by monitoring the oxidation of DHR 123. Briefly, 10 μl of skin homogenate was added to a rhodamine solution (50 mM sodium phosphate buffer, 90 mM sodium chloride, 5 mM diethylenetriaminepentaacetate [DTPA], and DHR 123). Changes in fluorescence intensity were measured every 5 min for 30 min on a fluorescence plate reader at excitation and emission wavelengths of 485 nm and 530 nm, respectively. 

To quantification of the end-products of lipid peroxidation, to be specific MDA, we performed thiobarbituric acid reactive substances (TBARS) assay in mouse skin homogenates. The 100 μl tissue samples were mixed in e-tubes containing 40 μl of 8.1% SDS solution, 300 μl of 20% acetic acid, and 200 μl of 1.2% thiobarbiturate solution. Tubes were heated in boiling water for 30 min, cooled to room temperature, 300 μl butanol was added, and mixtures were centrifuged at 1500 x g for 10 min. The absorbance of butanol layer was measured at 532 nm. Using MDA standard, TBARS were calculated as μM/mg protein.

### Statistical analysis

All results are expressed as means ± SEMs. Treatments were compared by one-way ANOVA followed by Dunnett’s test. Statistical significance was accepted for *P* values < 0.05. 
